# HyFormer: Hybrid Transformer and CNN for Pixel-Level Multispectral Image Land Cover Classification

**DOI:** 10.3390/ijerph20043059

**Published:** 2023-02-09

**Authors:** Chuan Yan, Xiangsuo Fan, Jinlong Fan, Ling Yu, Nayi Wang, Lin Chen, Xuyang Li

**Affiliations:** 1 School of Automation, Guangxi University of Science and Technology, Liuzhou 545006, China; 2 Guangxi Collaborative Innovation Centre for Earthmoving Machinery, Guangxi University of Science and Technology, Liuzhou 545006, China; 3 National Satellite Meteorological Center, China Meteorological Administration, Beijing 100081, China

**Keywords:** pixelwise classification, transformer, CNN, multispectral RS image classification

## Abstract

To effectively solve the problems that most convolutional neural networks cannot be applied to the pixelwise input in remote sensing (RS) classification and cannot adequately represent the spectral sequence information, we propose a new multispectral RS image classification framework called HyFormer based on Transformer. First, a network framework combining a fully connected layer (FC) and convolutional neural network (CNN) is designed, and the 1D pixelwise spectral sequences obtained from the fully connected layers are reshaped into a 3D spectral feature matrix for the input of CNN, which enhances the dimensionality of the features through FC as well as increasing the feature expressiveness, and can solve the problem that 2D CNN cannot achieve pixel-level classification. Secondly, the features of the three levels of CNN are extracted and combined with the linearly transformed spectral information to enhance the information expression capability, and also used as the input of the transformer encoder to improve the features of CNN using the powerful global modelling capability of the Transformer, and finally the skip connection of the adjacent encoders to enhance the fusion between different levels of information. The pixel classification results are obtained by MLP Head. In this paper, we mainly focus on the feature distribution in the eastern part of Changxing County and the central part of Nanxun District, Zhejiang Province, and conduct experiments based on Sentinel-2 multispectral RS images. The experimental results show that the overall accuracy of HyFormer for the study area classification in Changxing County is 95.37% and that of Transformer (ViT) is 94.15%. The experimental results show that the overall accuracy of HyFormer for the study area classification in Nanxun District is 95.4% and that of Transformer (ViT) is 94.69%, and the performance of HyFormer on the Sentinel-2 dataset is better than that of the Transformer.

## 1. Introduction

With its development, RS technology has come to play an important role in agricultural monitoring, geological surveys, disaster prevention and mitigation, and military investigations [1,2,3,4]. Land cover classification has been the focus and main challenge of RS technology, and land cover classification plays a great role in tasks such as precision agriculture, urban planning, geological change and mineral detection [5,6,7,8]. The purpose of RS image classification is to identify each pixel vector as a discrete set of specific classes [9], and researchers use the original spectral information or add NDVI, NDWI and other indices as the input. Support vector machines (SVMs) [10], the maximum likelihood estimation (MLE) [11], random forest (RF) [12], K-nearest neighbor (KNNs) [13] and other machine learning algorithms are proposed for RS image classification. The support vector machine classifies by seeking the optimal classification hyperplane, and random forest constructs multiple decision trees with randomly selected samples from the input pixel sequence to verify the unselected samples; and the final prediction result is obtained by voting. RS images contain not only spectral information, but also spatial information, such as shape and texture, so there are also RS image classification methods that combine spatial and spectral features [14]. The spatial feature extraction methods mainly include gray-level co-occurrence matrix [15], morphological filtering [16], Gabor filter [17], etc. Although adding spatial features is better than using single spectral features for classification, in the face of a complex ground environment, there are limitations to using spatial and spectral features, which cannot extract robust deep features well, and the classification results may not achieve the desired effect. Therefore, the traditional classifier still needs to be improved for the classification of refined agriculture.

Deep learning is a hot research area for the classification of fine-grained agriculture [18,19], which has gained wide applicability in the land classification task of RS images. The inputs of CNN applied to the land cover task can be broadly classified into three categories: fully annotated images, pixel blocks, and pixel sequences. Pixel point classification is performed on fully annotated images using a semantic segmentation network. A pixel block consists of the spectral information of the target pixel and the spectral information of its neighboring pixels. The 2D pixel block is classified using the network and its results are used as the classification result of the target pixel. The pixel sequence consists of the spectral information of the pixel and is classified using the network on a pixel-by-pixel sequence, thus enabling the classification of remotely sensed images. Semantic segmentation networks directly take the whole image as a sample, learn the features of RS images layer by layer, and output the segmentation results directly [20]. U-Net [21] is one of the most representative networks in semantic segmentation, and was first applied to medical image segmentation because U-Net can obtain good segmentation results even with fewer training data. Common U-Net networks and the improved networks based on U-Net include the following: John et al. [22] used Attention-UNet [23] to implement deforestation monitoring in the Amazon region and generated mask images using the K-means algorithm. Wei et al. [24] produced mask images based on samples collected outdoors and prior knowledge for the U-Net segmentation of rice in Northeast China. Su et al. [25] proposed RaftNet for segmenting aquaculture areas, which helps to accurately manage coastal aquaculture. Semantic segmentation has some advantages in areas with few feature types, and only image data from training sub-regions are needed to segment the whole target area. However, the production methods of mask images rely on traditional methods such as SVM, MLE, RF, KNN, etc. The quality of mask images may not be as good as desired in the face of complex environments, and the quality of mask images largely affects the generalization ability of the model. Meanwhile, land cover is difficult to classify; the images of the target area can be mapped into several or a dozen classes according to different applications, and it is difficult to reuse previous datasets.

To address the problem of mask image quality in complex imaging environments, as well as to increase the utility of deep learning for land cover classification, Hu et al. [26] proposed 1D CNN for the classification of pixel sequence inputs. One-dimensional CNN contains an input layer, a convolutional layer, a maximum pooling layer, a fully connected layer, and an output layer to classify hyperspectral images directly into the spectral domain. Since RNN [27] obtained good results when applied in challenging sequence data analysis, Mou et al. [28] considered that RNNs use a cyclic process to characterize the spectral correlation and band-to-band variability, and proposed a new RNN model with a special activation function; they improved the gated cyclic unit to analyze the hyperspectral pixels as sequence data, and then determined the information class using the network. Sidike et al. [29] proposed a deep progressive expansion network (dPEN) for the feature coverage analysis of WorldView-3 images using dPEN, which maps a total of 19 mapping categories and also explores the near-infrared band and short-wave infrared to improve the classification accuracy of images. dPEN’s input data are pixel vectors, and the output is the input pixel vectors corresponding to one of the categories. dPEN consists of three 1D-Conv layers, three maximum pooling layers, four progressive expansion layers, and one fully connected layer. Since graph convolutional networks can achieve pixel-level classification with better results, Ding et al. [30,31,32,33,34,35,36,37] focused on using graph neural networks to achieve hyperspectral image classification, solving the problems of graph neural networks in terms of multi-scale feature fusion and contextual information, and providing new ideas for the application of GCN in hyperspectral image classification. Although 1D CNN and RNN can achieve pixel-by-pixel classification and derive better results than traditional approaches, the network structure of CNN cannot handle the sequence properties of spectral features well [38]. With the great success of using Transformer [39] in the field of natural language processing (NLP), Transformer gradually replaced RNN as the mainstream feature extractor. The key to the success of Transformer is its superb sequence modeling capability and global information perception. Transformer architecture completely relies on Google’s Vision Transformer (ViT), established in 2020 [40], which successfully applied Transformer to image classification and triggered subsequent related research. ViT divides the input image into multiple patches, and then expands each patch into a sequence input. The pixel sequences of remote sensing images are natural sequence information, and the Transformer can make good use of pixel sequences to classify pixels. The SpectralFormer network was brought up in the [38], which rethought the RS image classification from the sequence perspective. The SpectralFormer was applicable to pixel-wise and patch-wise inputs, and can learn local spectral sequence information from neighboring bands to produce grouped spectral embeddings, and also transfer shallow information to deep layers via cross-layer skip connection.

The spectral–spatial feature approach has also been applied to deep learning, and the effective use of spectral–spatial information can improve the classification results, usually by dividing hyperspectral images into small chunks, each containing the spectrum and neighborhood of a specific pixel [41,42,43]. Makantasis et al. [44] used convolutional neural networks to encode a 5 × 5 resolution patch and used MLP for classification. Chen et al. [45] proposed a joint spectral–spatial classification framework, first using principal component analysis (PCA) to reduce the image dimensionality while preserving spatial information, extracting the neighborhood region of pixels and flattening it, and then fusing it with the original pixel information to achieve spectral–spatial information fusion. Hong et al. [46] used GCN to extract features of pixel-level input; CNN extracts the features of pixel blocks, and the extracted features are fused by additive fusion, element multiplication fusion, and cascade fusion to obtain the final features for classification. In contrast to the above methods of extracting spectral–spatial features, Chen et al. [47] proposed a 3D CNN approach for direct hyperspectral cube classification. Although many advanced deep learning algorithms try to improve the remote sensing image classification results via the patch-based approach instead of the pixel-based alternative, the patch-based approach assumes that the pixels neighboring the target pixels within a fixed spatial window belong to this same class—an assumption that is not always true—and this problem will be more prominent if the remote sensing data are of lower resolution [48]. Among the publicly acquired satellite data, the most commonly used are LandSat 8, with a 30 m resolution, and Sentinel-2, with a 10 m resolution. Depending on the classification task, different satellite data are selected, and multispectral data such as Sentinel-2 and LandSat 8 are suitable for large-scale land cover classification, although some non-publicly acquired hyperspectral data have a higher resolution. However, the cost of data acquisition is excessively high for most scientific research and practical projects. To solve this problem, it is only necessary to convert 1D pixel spectral sequences into 3D spectral feature matrices, turning the spectral sequence information problem into a feature matrix classification problem. This also makes the spectral sequences applicable to most 2D CNNs, improving the application scope of network frameworks in remote sensing classification, and avoiding the problem that CNN does not process sequence information and the arising of a situation in which neighboring pixels do not necessarily belong to the same class of problem, especially for high-resolution images with complex features. Another issue is that the CNN’s own backbone network is limited and has an insufficient ability to characterize the attributes of pixel sequences, while Transformer is good at analyzing sequence data. Adopting Transformer to process pixel sequence information is an effective approach.

In this paper, a new multispectral image pixel-by-pixel classification network framework, called HyFormer, is proposed to address the above problems. HyFormer adopts a serial network structure, taking into account the advantages of both CNN and Transformer. This paper revisits the multispectral image classification problem from the perspective of 1D pixel sequence information, and the main contributions are as follows:

(1) A shallow CNN structure is redesigned to achieve the 2D CNN classification of pixel sequences. The 1D pixel sequences are transformed into 3D spectral feature matrices so that they can be applied to most 2D CNNs, and the generalizability of 2D CNNs for 1D-pixel sequence classification is expanded;

(2) A framework for fusing CNN and Transformer for the pixel-level classification of multispectral images is proposed. The three levels of features extracted using CNN are transformed into sequence data as part of Transformer’s input. Feature fusion between neighboring encoders is achieved by skip connection, allowing effective features to be passed to deeper layers;

(3) The dynamic land use change analysis of Changxing County data focuses on the changes in pond and rice distribution areas during the period 2018–2022, because pond and rice are the local pillar industries.

The rest of the paper is organized as follows: Section 2 introduces the materials and methods, Section 3 presents the results in detail, Section 4 develops the discussion, and Section 5 outlines conclusions.

## 2. Materials and Methods

### 2.1. Study Area Overview

**Figure 1:**
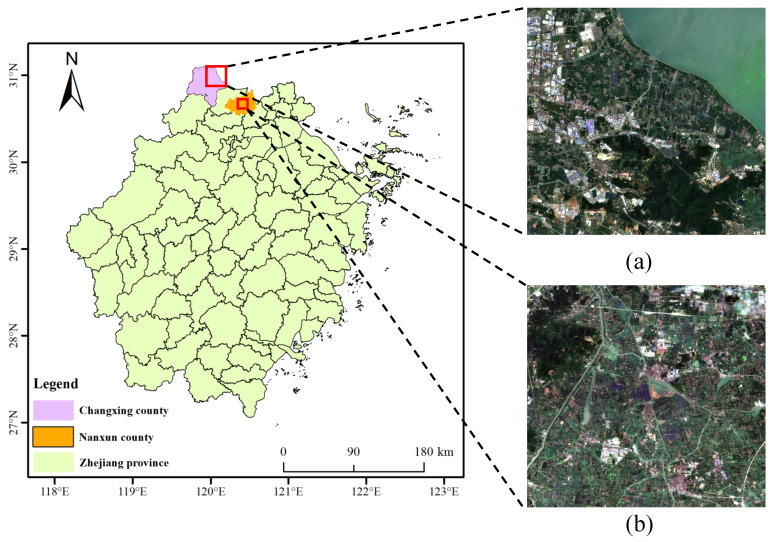
Location of Changxing County and Nanxun District, and Sentinel-2 remote sensing images. (**a**) Changxing County; and (**b**) Nanxun District.

Changxing County is part of Huzhou City, Zhejiang Province, China, between 30°43′–31°11′ N latitude and 119°33′–120°06′ E longitude, located in the Hangjiahu Plain of the Yangtze River Delta, southwest of Taihu Lake, the third largest freshwater lake in China. The rich water resources have created a good environment for local farmers to develop aquaculture, and the main elements of aquaculture are river crabs, crayfish, bullfrogs, etc. The study area (as shown in Figure 1) contains more river crab farming areas (pond), and it is important to obtain the crab pond area accurately and effectively in order to carry out the development of local aquaculture and yield estimation.

Nanxun District is located in the eastern part of Huzhou City, Zhejiang Province, China, in the southern part of Taihu Lake, with similar geotypes to Changxing County, and has abundant aquatic and agricultural crops.

### 2.2. Field Sampling and Remote Sensing Image Preprocessing

To obtain the distribution of actual feature types in the study area, field sampling was conducted in June 2021 to obtain the sample points of different feature types through field data collection and field observations. In the process of selecting the samples of crops and aquatic areas, priority was given to the contiguous planting areas with an area larger than 100 m^2^, and the acquired data were used to accumulate a priori knowledge and later perform accuracy verification. On the one hand, the data we collected were sampled in large planting areas, and the geographical location of the corresponding sample points was recorded during the sampling process. These samples (forests, cities, crab ponds, rice, plantation, and greenhouses) are the main economic pillar industries of the local area, and all have previous inputs, so the planting patterns are relatively fixed and are generally planted continuously for several years, with little change from year to year. On the other hand, when generating the 2022 sample, the geographic location information collected in 2021 was combined with higher resolution images (e.g., Google Maps, unmanned aerial vehicles remote sensing images, etc.), and the crop growth patterns from different time series images were combined with expert a priori knowledge to determine the categories of selected features to ensure accurate sample information.

**Figure 2:**
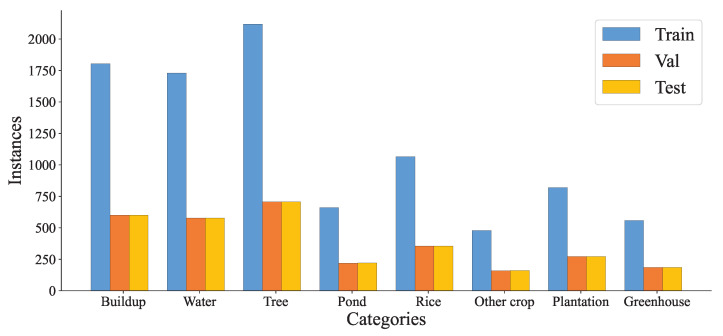
Class-label instances for Changxing County sample bank.

**Figure 3:**
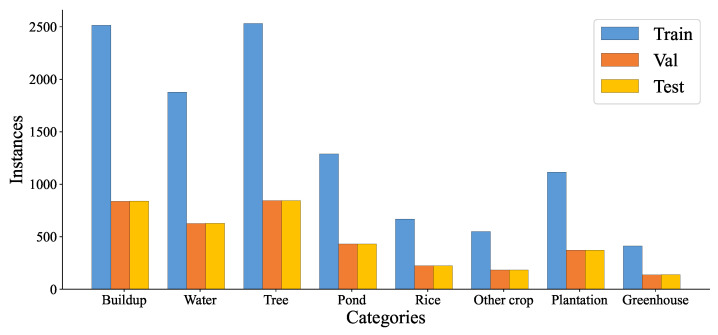
Class-label instances for Nanxun District sample bank.

In this paper, a multispectral image covering the study area taken by the Sentinel-2B satellite with a resolution of 10 m on 27 July 2022, which contains 13 bands, was used as the data source. The Level 1C data were first radiometrically calibrated, and then atmospherically, topographically, and cirrus-corrected using Sen2Cor software to improve the scene quality. Then, the SNAP software was used to resample the non-10 m resolution band resolution to a 10 m resolution, which facilitates the generation of band files in the img format for band synthesis, and finally ENVI software was used to select Band2, Band3, Band4, and Band8 (bands with an original resolution of 10 m, RGB + NIR) for band synthesis and export the images in tiff format. Then, a sample library of data for Changxing County was formed from field data and a priori knowledge, using ENVI software to manually mark ROI based on latitude and longitude information obtained from outdoor sampling. The sample library contains 15,391 samples in 8 categories: 60% of each category was used for training, 20% for validation, and 20% for testing. The categories and number of samples are shown in Figure 2. Data were obtained from the Nanxun district sample bank. The sample library contains 18,263 samples in 8 categories; 60% of each category was used for training, 20% for validation, and 20% for testing. The categories and numbers of samples are shown in Figure 3.

### 2.3. HyFormer

**Figure 4:**
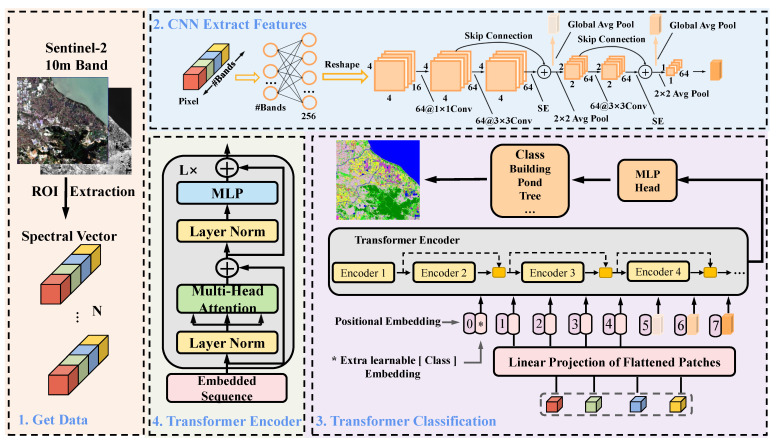
Schematic diagram of HyFormer architecture for the multispectral image classification task. The HyFormer consists of an FC, CNN, feature fusion module, and Transformer.

The HyFormer architecture proposed in this paper for 1D pixel sequence classification is shown in Figure 4. HyFormer consists of an FC, CNN, skip connection, and Transformer. The first step sends a multispectral pixel sequence to the FC, adjusts the dimensionality of the 1D spectral sequence using the linear transformation function of the fully connected layer, and reshapes the transformed 1D spectral sequence into a 3D spectral feature matrix. The second step extracts the features of three different levels of 2D CNN as part of the Transformer’s input. The third step combines the CNN-extracted features with the position encoding together with the pixel information while adding the position encoding. The fourth step achieves feature fusion by skip connection between neighboring Transformer encoders. Finally, MLP head obtains the classification results and achieves pixel-level classification.

#### 2.3.1. Feature Fusion Module

Skip connection (SC) enables previously learned features to be used in deeper layers, enabling the fusion of multiple features and reducing the loss of information. Skip connection has been shown to be an effective method in classical networks, such as ResNet [49] and U-Net [21]. However, the large difference in features between long skip connection may cause the effective features to be interfered, which is not good for classification, so short skip connection and medium-distance skip connections are mainly considered in the Transformer. SpectralFormer [27] is a cross-layer adaptive fusion (CAF) method, while CAF is a medium-distance skip connection strategy. CAF skips one encoder and fuses two layers of features one encoder away from each other. Unlike CAF, we use short hopping links to achieve feature fusion, which makes full use of the features between adjacent layers and reduces the information loss. The feature fusion module, shown in Figure 4, splices the features of the previous encoder $\left(E^{i-1}\right)$ with the features of the current encoder $\left(E^{i}\right)$$\left(Concat\left(E^{i-1},E^{i}\right)\right)$, and then changes the dimensionality through a convolution layer $\left(Conv\left(Concat\left(E^{i-1},E^{i}\right)\right)\right)$ to obtain the features after fusion. It can be expressed as:

(1) $$\hat{E}=Conv\left(Concat\left(E^{i-1},E^{i}\right)\right)$$

where $E^{i},E^{i-1}\in R^{n\times dim}$ is the dimension of the extension $E^{i}$, $E^{i-1}\in R^{n\times dim\times 1}$, $Concat\left(E^{i-1},E^{i}\right)\in R^{n\times dim\times 2}$, and the convolution kernel of conv is [1, 2], $\hat{E}\in R^{n\times dim}$.

#### 2.3.2. Fully Connected Layer with 2D CNN

The multispectral image can be represented as $X={\left\{x_{i}\right\}}_{i=1}^{H\times W\times 1}\in R^{H\times W\times 1}$, where H and W denote the height and width of the multispectral image, C denotes the number of bands of the multispectral image, and $x_{i}\in R^{1\times 1\times C}$ denotes the i-th pixel sequence. The 1D pixel sequences are not processed in any way and can only be classified by 1D CNN. If a 2D CNN is used for classification, the 1D sequence must be converted to a 2D/3D spectral feature matrix, or a patch of central pixels must be used as a sample. In this study, a fully connected layer is utilized to boost the dimensionality of the 1D pixel sequence and then reshape it into a 3D pixel feature matrix for use as the 2D CNN input.

(2) $$y_{i}=wx_{i}+b$$

where *w* and *b* represent the weight matrix and bias vector of the fully connected layer, respectively, $x_{i}$ is the input 1D pixel sequence, $y_{i}\in R^{1\times 1\times m}$, and m is the output dimension of the fully connected layer and reshapes $y_{i}$ as $y_{i}^{\prime }$, $y_{i}^{\prime }\in R^{n\times n\times \frac{m}{n^{2}}}$; *m* = 256 and *n* = 4 in this paper.

**Figure 5:**
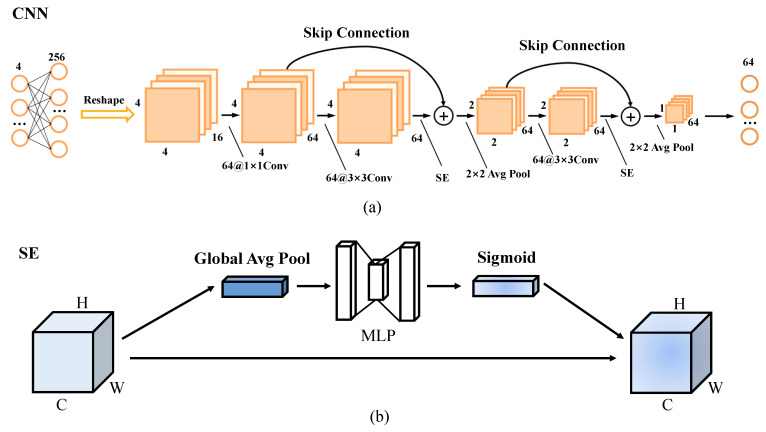
(**a**) Overall structure of 2D CNN; and (**b**) SE module.

Since Transformer training takes more time, a shallow 2D CNN is redesigned with the network structure shown in Figure 5. Although the network structure is simple and the computation required is small, it can also achieve multispectral image classification. The first convolutional layer contains 64 convolutional kernels, and the size of the convolutional kernels is 1 × 1. The first convolutional layer is used to enhance the dimensionality of the input samples, the second convolutional layer contains 64 convolutional kernels, and the size of convolutional kernels is 3 × 3. The second convolutional layer is connected with an SE module [50] after the second convolution is used. The concern is developing more effective feature information, and the first convolutional layer and the second convolutional layer are formed into a residual unit through residual connection. The residual unit is connected to a 2 × 2 averaging pooling layer to reduce the redundancy of information and expand the perceptual field. After averaging pooling is applied to the two residual units, the features are squashed into a vector form. The features after each residual unit are extracted and the extracted features (n×n×m and n/2×n/2×m) are converted into serial data (1×1×64) using a global averaging pooling operation. $y_{i}^{\prime }\in R^{n\times n\times \frac{m}{n^{2}}}$ is the input to the CNN, *n* = 4, *m* = 64, and the output of the CNN is 1×1×64.

#### 2.3.3. Transformer

**Figure 6:**
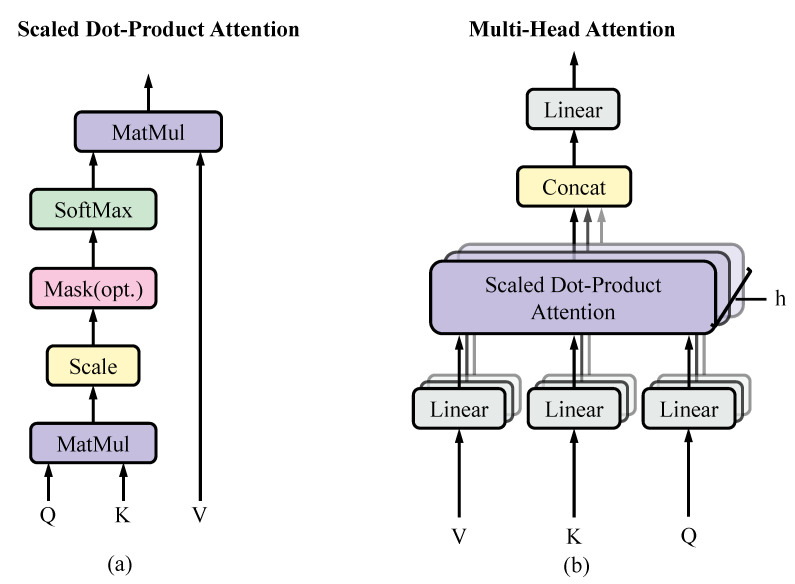
(**a**) Scaled dot–product attention. (**b**) Multi-head attention consists of several attention layers running in parallel.

Transformer is mainly used to deal with sequence-to-sequence problems and was originally used in NLP. Due to its use of a self-attentive mechanism and positional coding, Transformer’s sequence-to-sequence transformation is different from that of RNN, and does not need to keep the length of input and output sequences consistent, which has great advantages for processing sequence data. The structure of Transformer enables it to obtain adaptive spatial information and can establish long-term interdependencies between sequences. Transformer has shown good performance in image classification, semantic segmentation and other image processing-related fields [40,51,52,53]. In particular, ViT shows good performance in image classification, which promotes the development of Transformer in the field of image processing. The Vision Transformer used in this paper uses the Transformer Encoder (shown in Figure 4) as an encoder, and a single Transformer Encoder consists of three parts: input, attention mechanism, and feedforward neural network. The input part involves position encoding and input feature embedding. Transformer establishes long-term interdependencies that depend on location encoding, while the association between important items of information is achieved based on the self-attentive mechanism. The self-attention mechanism is also called multi-headed attention, as shown in Figure 6. The pixel sequence in the Transformer (ViT) can be implemented via the four following steps, As shown in Algorithm 1.

Step 1: Input sequence data *x*, and m is the number of bands. We divide this into m vectors, and pull up linearly for each vector to obtain a new *x*.

Step 2: Add the location code, add the location encoding information to *x* to obtain the new *x*.

Step 3: Pull up the dimension to 3×dim by linear transformation, in order to implement the multi-head approach, Q,K,V are reshaped and normalized according to the number of heads, which are used for the input of self-attentiveness after normalization.

Step 4: Take a head as an example, compute Q and K in the form of inner product, divide the result of the obtained inner product by $\sqrt{dim}$, implement the activation function on Softmax, and finally multiply it by V to obtain the attention $Z_{i}$.

(3) $$Attenion\left(Q,K,V\right)=\mathrm{softmax}\left(\frac{QK^{T}}{\sqrt{d_{k}}}\right)V$$

**Algorithm 1** Multi-Head attention.**Input:** X 1D spectral band information $\left(x=\left[x_{1},x_{2},\ldots ,x_{m}\right],x_{i}\in R^{1\times 1},i=1,2,\ldots ,m\right)$**Output:** Z
1:Generate group-wise spectral embedding $\left(x=\left[x_{1},x_{2},\ldots ,x_{m}\right],x_{i}\in R^{1\times dim},i=1,2,\ldots ,m\right)$2:Add positional embedding $\left(x=\left[x_{0},x_{1},x_{2},\ldots ,x_{m}\right],x_{i}\in R^{1\times dim},i=0,1,\ldots ,m\right)$3:Generate Q, K, V $\left(Q,K,V\in R^{h\times \frac{dim}{h}\times \left(m+1\right)},Q=\left[Q_{0},Q_{1},\ldots ,Q_{m}\right],K=\left[K_{0},K_{1},\ldots ,K_{m}\right],V=\left[V_{0},V_{1},\ldots ,V_{m}\right]\right)$4:Multi-head attention5:**output**: Z-generated attention representation $\left(Z=\left[Z_{1},Z_{2},\ldots ,Z_{h}\right]\in R^{h\times \frac{dim}{h}\times \left(m+1\right)}\right)$


### 2.4. Evaluation Metrics

For the evaluation of the classification of multispectral pixels, three evaluation metrics based on the confusion matrix are used, including accuracy (the ratio of the number of correct predictions to the number of all predictions), precision (the ratio of correct predictions as positive classes to all predictions as positive classes), and Kappa coefficient (a measure of the consistency of two variables). The formula for quantitative assessment is as follows:

(4) $$Accuracy=\frac{TP+TN}{TP+FN+FP+TN}$$

(5) $$Precision=\frac{TP}{TP+FP}$$

(6) $$Kappa=\frac{p_{o}-p_{e}}{1-p_{e}}$$

where *TP* is the classified accurate positive class, *FP* is the misclassified positive class, *TN* is the classified accurate negative class, and *FN* is the misclassified negative positive class. $P_{o}$ is the overall precision, and $P_{e}$ is the chance compliance rate.

### 2.5. Loss Function

Due to cross entropy can better handle multi-categorization tasks [54], it is chosen as the loss function for multi-classification tasks. The cross-entropy loss function compares the predicted class with the target class for each pixel, and its expression is as follows:

(7) $$L=-\frac{1}{N}\sum_{i}\sum_{c=1}^{M}y_{ic}\log\left(p_{ic}\right)$$

where *M* denotes the number of categories and $y_{ic}$ is the sign function (0 or 1). If the true category of sample *i* is equal to *c*, take 1, otherwise take 0; $p_{ic}$ is the predicted probability that the observed sample *i* belongs to category *c*.

### 2.6. Experimental Environment

In the experiments, the sample points and the learned interest marker interest regions were obtained by outdoor sampling using the ENVI software, and the coordinates of the interest regions are derived. In the training phase, adam [55] is chosen as the optimizer, the minbatch size is set to 32, the maximum number of iterations is 300, and after literature research and experimental validation, 5 × 10^−4^ was finally chosen as the initial learning rate; every 30 iterations, it is multiplied by a factor decay of 0.9. All the implementation code is implemented by Python 3.7 in pytroch1.12.1, and the training of the model is undertaken in win11 + AMD Ryzen 9 5900HX + NVIDA GeForce RTX 3080 Laptop GPU.

## 3. Result

The details of the sample pool in the study area are shown in Table 1, with 60% used for training, 20% for validation, and 20% for testing. The following experiments were conducted using the data in Table 1. Due to the uneven distribution of samples, we have tried to proportionally give more attention to less represented classes during training; however, there is a negative impact on accuracy and therefore no weights are added to the loss function.

To highlight the effectiveness of our model, HyFormer is compared with SVM [10], 1D CNN [26], RNN [27], Transformer [39], and SpectralFormer [38] in this paper. The specific parameters are shown in Table 2.

(1) For the SVM, the kernel function uses the radial basis function (RBF), the penalty factor is set to 10, and the decision function shape is set to “ovr”.

(2) For the 1D CNN, a convolutional layer, a batch normalization layer, a ReLU activation layer, a maximum pooling layer, a fully connected layer, and an output layer are included.

(3) For the RNN, there are two recurrent layers with a gated recurrent unit.

(4) For the Transformer, the structure of ViT [39] is used, with five encoder blocks, grouped spectral embedding of dimension 64, and each encoder block contains four self-attentive layers and eight hidden layers of MLPs and dropout layers that suppress 10% of neurons.

(5) For the SpectralFormer, there are five encoder blocks; each encoder block contains four self-attentive layers, eight hidden layers of MLPs and dropout layers that suppress 10% of neurons. The dimension of each grouped spectral embedding is 64, and the grouped spectral embedding is set to 2.

(6) For the FC + 2D CNN, the input dimension of the fully connected layer is set to 4, the output dimension is 256 and the reshape is (4, 4, 16) features. The first convolutional layer contains 64 convolutional kernels with a size of convolutional kernels of 1 × 1, and the second convolutional layer contains 64 convolutional kernels with a size of convolutional kernels of 3 × 3, after which an SE module is connected, and the first convolutional layer and the second convolutional layer are formed into a residual unit by residual connection; the residual unit is followed by a 2 × 2 average pooling layer, and there are two residual units.

(7) For the HyFormer, the Transformer (ViT) module contains a total of five encoder blocks, each containing four self-attentive layers, eight hidden layers of MLPs and dropout layers that suppress 10% of neurons.

### 3.1. Learning Rate Selection

Since the learning rate has a large influence on the classification results, in order to select a suitable learning rate, Hyformer was used as the main backbone network, the Changxing County study area was used as the dataset, and initialized learning rates of 0.05, 0.005 and 0.0005 were selected for the experiments. The experimental results are shown in Table 3. When lr = 0.0005, the classification accuracy was the highest, so 0.0005 was chosen as the initialized learning rate.

### 3.2. Ablation Study

To evaluate the performance of the network structure proposed in this paper, ablation experiments were performed on the dataset in the study area. The experimental results are shown in Table 4. The OA of the original ViT is 94.15%, which is a satisfactory result, indicating that ViT is well suited to handling multispectral image classification problems. The OA of FC + CNN is 94.41%, which is 0.26% higher than that of the original ViT (94.15%), although the OA of FC + CNN is higher than that of ViT, proving the effectiveness of the FC + CNN scheme, this can be achieved using the fully connected layer. After changing the dimension of the pixel spectral sequence, it is reshaped into the input feature size required by 2D CNN, and the classification of 1D pixel sequences by 2D CNN is achieved. Using the features extracted by 2D CNN as part of the Transformer input, the OA of this structure is 95.27%, which is 1.12% higher than ViT. Thus, it is verified that CNN + ViT is more advantageous for multispectral image pixel classification tasks compared to ViT. The addition of the skip connection brings some improvements to ViT, and the OA after adding the skip connection is 94.25%, which is 0.1% higher than ViT. The OA of HyFormer is 95.37%, which is 1.22% higher than that of ViT. Both strategies, either individually integrated with ViT or together, improve the performance of ViT.

**Figure 7:**
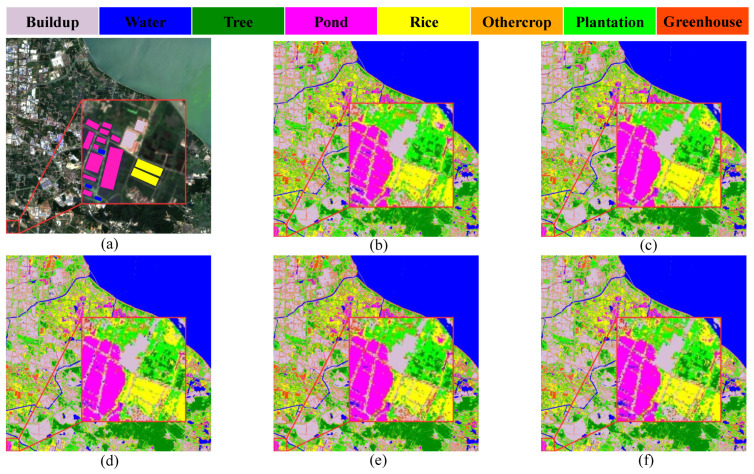
Plot of the classification results of the Changxing County’s dataset using HyFormer with different module combinations: (**a**) Image; (**b**) Transformer (ViT); (**c**) FC + CNN; (**d**) HyFormer (ViT + CNN); (**e**) HyFormer (SC); and (**f**) HyFormer (ViT + CNN + SC).

The classification results of different module pair combinations on the study area’s dataset are shown in Figure 7. The red boxes in Figure 7a primarily contain pond, rice, buildup and a small amount of water. Comparing the observations in terms of details, HyFormer works better than a single module with a ViT structure in terms of details. Since the region of pond may contain less water, resulting in water being easily misclassified as pond, ViT and ViT+SC are able to more effectively distinguish pond and water, while 2D CNN and 2D CNN + ViT are less effective, and HyFormer is the best. HyFormer includes less noise in its classification results for buildup. The classification results are similar for both rice regions.

**Figure 8:**
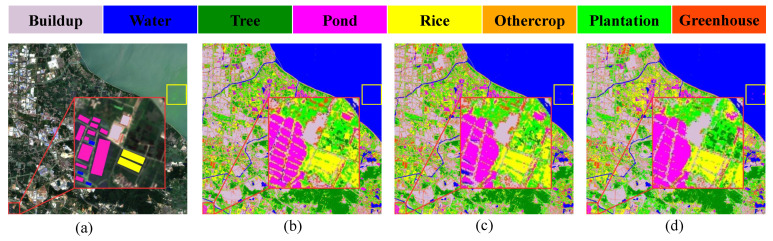
Plots of training sample results with different ratios: (**a**) Original image; (**b**) 10%; (**c**) 20%; and (**d**) 40%.

In order to evaluate the effect of using fewer training samples on the experimental results, we randomly selected 10%, 20%, and 40% of the samples from the sample pool of Changxing County for training and the remaining samples were used for testing without a validation set. The experimental results are shown in Table 5 and Figure 8. As the training samples increase, the ratio of classification samples increases, the OA gradually rises, and less noise is generated. However, because random sample selection leads to random sample point selection, randomly selected training sample points may not contain all sub-regions of the ROI, resulting in higher test accuracy, but this will generate obvious misclassifications, such as the yellow boxes in (c) and (d), which misclassify water as pond. As such, in order to achieve the best overall classification, we chose a ratio of 6:2:2 for the experiment.

### 3.3. Comparison of Multiple Methods

The quantitative classification results of OA, AA, and kappa for the study area’s dataset and the accuracy of each category are shown in Table 6 and Table 7. From Table 6, it can be seen that 1D CNN has the worst overall effect, the accuracies of OA, AA, and kappa are lower than those of other models, and the accuracies for the pond, other crops and greenhouse are only 57.27%, 10.69%, and 54.84%, respectively. The OA of the traditional classifier SVM is 89.31, which enables slightly worse classification results, and SVM has a poor classification ability for other crops and the best classification effect for trees. RNN, ViT, SpectralFormer, FC + 2D CNN and HyFormer are all deep learning-based spectral sequence classification methods, the OA of RNN is 93.58%, the OA of ViT is 94.15%, the OA of SpectraFormer is 93.87%, and the OA of FC + 2D CNN is 93.58%. RNN achieves the best classification for the greenhouse: ViT and SpectralFormer achieve better segmentation for rice; ViT, SpectralFormer, and HyFormer achieve the same classification accuracy for pond; HyFormer has the same classification accuracy for buildup and higher for water and other crops. Buildup (+3.07%), water (+0.3%), other crops (3.77%), OA (+1.22%), AA (1.07%), and Kappa (+0.0144) are all improved compared with ViT. Interestingly, the method of FC + 2D CNN proposed in this paper also achieves better results. As seen in Table 7, FC + 2D CNN and HyFormer still a achieve higher OA than this algorithm, and are more advantageous when used in classifying the study area.

**Figure 9:**
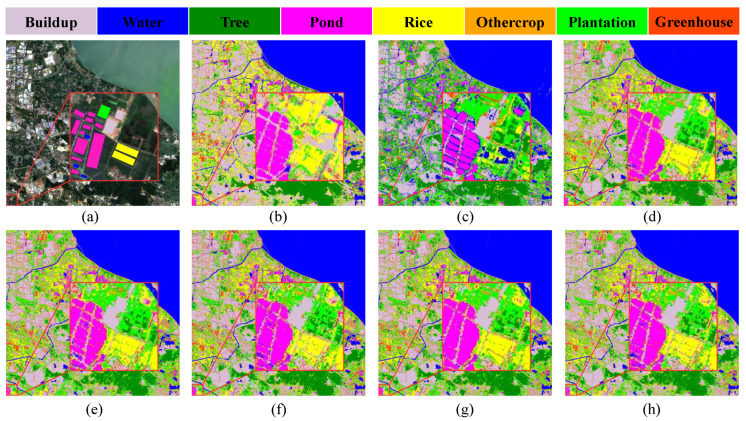
Classification results obtained by different models applied on Changxing County data. (**a**) Image; (**b**) SVM; (**c**) 1D CNN; (**d**) RNN; (**e**) Transformer (ViT); (**f**) SpectralFormer; (**g**) FC + 2D CNN; and (**h**) HyFormer.

**Figure 10:**
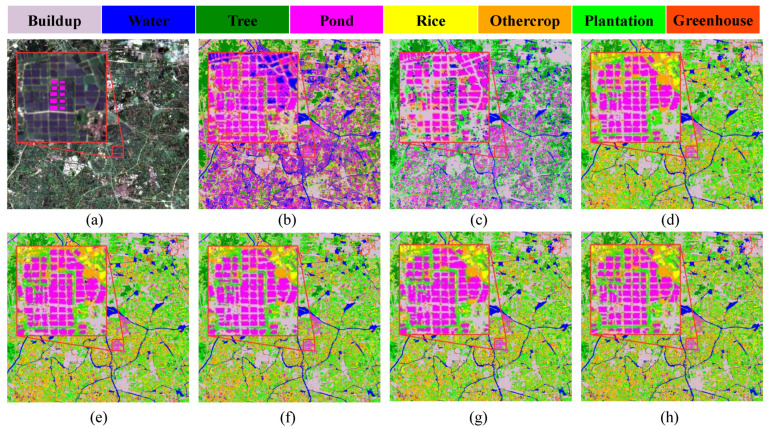
Classification results obtained by different models applied on Nanxun district dataset: (**a**) Image; (**b**) SVM; (**c**) 1D CNN; (**d**) RNN; (**e**) Transformer (ViT); (**f**) SpectralFormer; (**g**) FC + 2D CNN; and (**h**) HyFormer.

The classification map of Changxing County obtained by different models is shown in Figure 9. According to the a priori knowledge as well as outdoor sampling data, some areas in other boxes are marked, and the red box in Figure 9 primarily contains the five categories of buildup, pond, rice, water, and plantation. SVM is not able to easily detect rice and plantation, which obviously produces confusion. The overall classification results of RNN, ViT, SpectralFormer, FC + 2D CNN, and HyFormer are better, but the results of HyFormer are better locally. HyFormer can better distinguish between ponds and plantations with small intra-group differences, and between ponds and water with less difference. The pond in the red box contains three regions of water, while ViT, SpectralFormer, and HyFormer extract only two regions of water, but the results of HyFormer are clearer than those of the other two algorithms. As seen in Figure 10, the overall classification results of SVM and 1D CNN differ from the actual scenario and the results of the remaining algorithms. HyFormer extracted the edges of ponds more clearly and chunked them more obviously, and the regions between adjacent pond areas were not misclassified as pond. In general, 2D CNN, RNN, and ViT performed significantly better than 1D CNN and SVM. The proposed FC + 2D CNN achieved higher OA than ViT and required a shorter training time. HyFormer required a slightly longer training time than ViT, but its OA, AA, and kappa showed some improvement, and the image formation effect was also better than that of ViT, which makes it worthwhile.

### 3.4. The Impact of the Sentinel-2 Red Edge or SWIR Bands on Land Cover Classification

In order to investigate the influence of the red edge or SWIR bands on feature classification, Changxing County was used as the study area, and the red edge or SWIR bands were added to RGB + NIR for experimental analysis, respectively. The results of the study area test set experiments are shown in Table 8 and Table 9. Before the addition of NIR and SWIR bands, the accuracy of the method proposed in this paper was higher than the other compared methods. After the addition of NIR and SWIR, respectively, the accuracy of the deep learning methods were improved, which is meaningful for the land cover classification task; however, the improvement of the method in this paper is not very large compared with other methods, and the other algorithms achieved good classification results.

### 3.5. Land Use Change in Study Area

In this paper, data from the USGS website (https://earthexplorer.usgs.gov/) were downloaded for 9 May 2018, 1 August 2020, and 27 July 2022, specifically the Sentinel-2 series images, in order to carry out the land cover classification of Changxing County. The first step was to select the ground samples. The sample selection process involved field sampling in 2021 across a large area of cultivation, with information on the geographical location of each sample point recorded during the sampling process. These samples (forests, cities, ponds, rice, plantation, and greenhouses) are mainly the main economic pillars of the local area, and all have previous inputs, so the cropping pattern is relatively fixed, and they are generally planted continuously for several years, with little variation from year to year. On the other hand, when generating samples for other years, the geographic location information collected in 2021 was combined with expert a priori knowledge and high-resolution imagery (e.g., Google, unmanned aerial vehicles remote sensing image) to generate sample data for other years (2018, 2020). Finally, different models were trained based on the samples selected from each of the three different years and the remote sensing images from the corresponding years were classified. In the classification process for each year, the training set, testing set and validation set were randomly divided into a 6:2:2 ratio, and the sample database data of 2018, 2020 and 2022 are shown in Table 10. In order to better compare each model, in this paper, the models in Table 6 have also been used to compare and analyze the different methods after the training of the data from the three images, and finally, the HyFormer proposed in this paper was used to carry out land use change analysis of the study area, focusing on the changes in the areas of pond and rice.

The classification results of different models for the sample pool data of 2018, 2020, and 2022 are shown in Table 6, Table 11 and Table 12. The OA values of HyFormer were 94.87%, 96.15% and 95.40% in the datasets of three different years, which were higher than the other models, and improvements of 2.16%, 0.87%, and 1.22% compared to the original ViT, respectively. The pond classifications for 2018 and 2022 were also better than those of other models. The classification performance of HyFormer meets the requirements for conducting land use change analyses of the study area.

**Figure 11:**
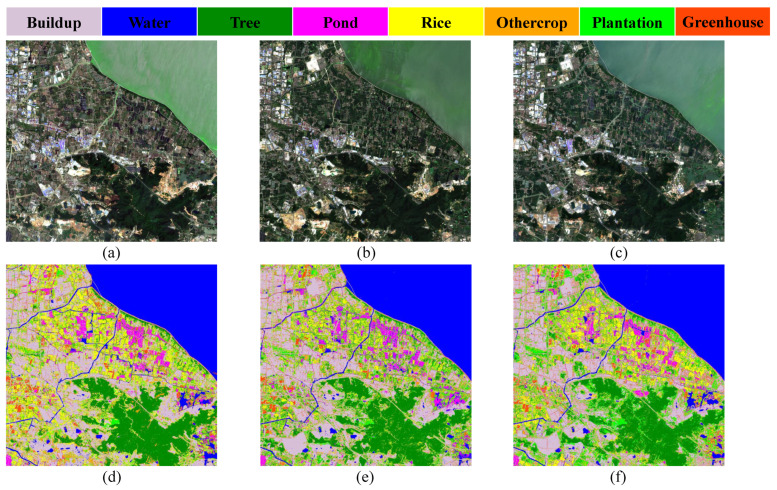
True color maps of the three images (**a**) 2018, (**b**) 2020; and (**c**) 2022; spatial distributions of features in the three images (**d**) 2018; (**e**) 2020; and (**f**) 2022.

From the evaluation indexes of 2018, 2020, and 2022, it can be seen that HyFormer achieves the best classification results for the three images, so the algorithm in this paper is used to carry out the dynamic monitoring of feature classification in the study area for the past four years. The spatial distributions of land use in 2018, 2020, and 2022 are shown in Figure 11, and it can be observed that the spatial distribution of each feature is more concentrated; specifically, pond areas are concentrated near Taihu Lake and rivers. The rich water resources promote the development of aquaculture and rice cultivation in the area around Taihu Lake. Greenhouses are scattered in the rice cultivation area.

The land use and land use change rate of the study area in the last 4 years are shown in Table 13. In the three images, buildup covers the largest proportion of the study area, followed by water and trees. Trees and water show less change in the last 4 years; most of the water area is located in Taihu Lake, but some is in rivers or reservoirs, and trees are mainly concentrated in the southeast area of the study area, with less change. During the 2018–2022 period, the area of buildup decreased instead of increasing because the bare land near the buildup was also marked as buildup in this paper when selecting ROI, so the decrease in buildup may be due to the conversion of bare land into other planting areas. At the same time, the Chinese government has started to regulate illegal construction in recent years, and some illegal buildings have been demolished and turned into other areas, which has led to the decrease in buildup. The crop cultivation areas include rice, other crops, and greenhouses. During the four-year period, the cultivation area of rice decreased and then increased, the area of other crops gradually decreased, and the area of greenhouse increased year by year.

**Figure 12:**
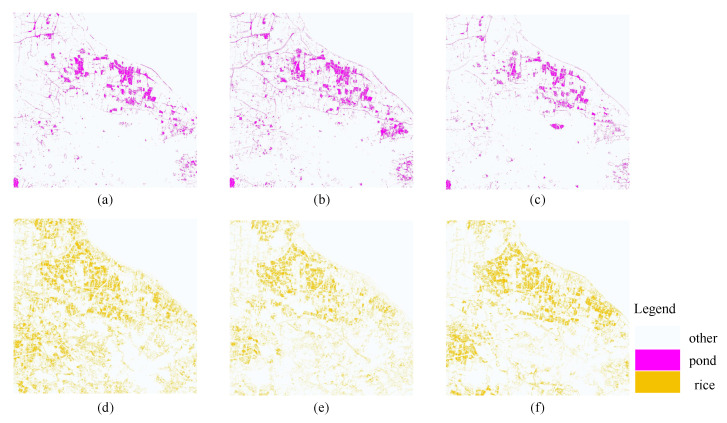
Spatial distribution of ponds in the three images: (**a**) 2018, (**b**) 2020, and (**c**) 2022; spatial distributions of rice in the three images: (**d**) 2018, (**e**) 2020, and (**f**) 2022.

Since ponds and rice are the main aquatic species and crops in the study area, this paper focuses on their spatial distributions; the spatial distribution map is shown in Figure 12, and the classification color of rice is enhanced for easy observation. From the overall observation, we can see that the area where ponds are mainly located is adjacent to the left bank of Taihu Lake, and the rest of the pond area is scattered near the river. The abundant water resources in Taihu Lake are conducive to the development of aquaculture as well as planting, and the high price of crabs has encouraged local farmers to vigorously develop farming. Rice is distributed in various areas of the study area, and in 2019, Daxing County introduced aerospace rice seeds. Aerospace rice has a higher production value and better taste than ordinary rice, so local rice planting was increased to maximize the land benefits.

**Figure 13:**
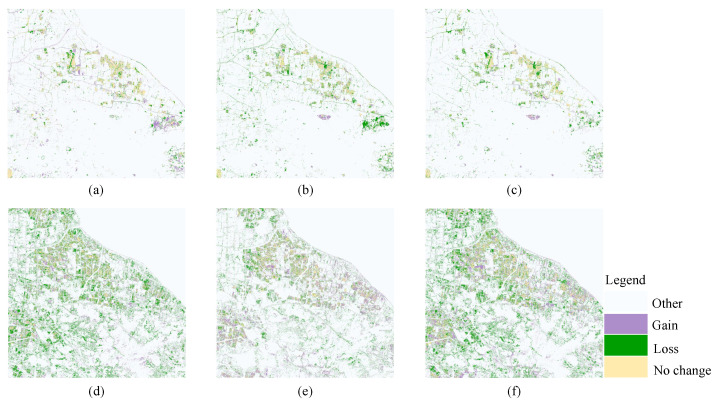
Dynamics of ponds in 2018–2020 (**a**), 2020–2022 (**b**), and 2018–2022 (**c**); dynamics of rice in 2018–2020 (**d**), 2020–2022 (**e**), and 2018–2022 (**f**).

The dynamic changes in pond and rice areas from 2018 to 2022 are shown in Figure 13; the area of pond in 2018 was 10.6323 km^2^, 13.3764 km^2^ in 2020 and 8.3785 km^2^ in 2022, and the farming areas in 2018–2020, 2020–2022, and 2018–2020 increased by 25.81%, decreased by 37.36% and decreased by 21.20%, respectively. The overall trend showed a decrease from 2018 to 2022 as the cost of raising crabs gradually increased; the retail price of crabs, although considerable, was unstable, leading to a decline in farmers’ income, while as the temperature gradually rises, crab’s shelling rate slows, their sizes become small, they show high mortality, and farmers move away from crab farming. The rice planting area in 2018 was 32.6588 km^2^, in 2020 it was 21.5091 km^2^, and in 2022 it was 25.3542 km^2^; the trends were decreasing by 34.14%, increasing by 17.88% and decreasing by 22.37% in 2018–2020, 2020–2022, and 2018–2020, respectively. The large changes in rice cultivation area, especially in 2018–2020, may be due to local farmers or contractors using the land for greenhouse vegetable cultivation for greater profit; as can be seen from Table 11, the area of greenhouses gradually increased. However, as the new high-yielding rice seeds started to become popular, farmers and contractors again invested in the cultivation of rice, so the area of rice cultivation was expanded again during the period 2020–2022.

## 4. Discussion

In the feature classification of Sentinel-2 multispectral images, only four 10 m bands (RGB and NIR) were selected from the 13 bands for pixel sequence information, because many satellites contain RGB and NiR bands but not necessarily others, and to increase the generality of the model, we sought to select only common bands. In the study area’s dataset, the traditional method SVM, and the deep learning methods 1D CNN, RNN, Transformer (ViT), SpectralFormer, FC + 2D CNN, and HyFormer, showed different performances. The advantage of SVM is its fast training time, while its disadvantage is that its sensitivity to sequence information is not as good as RNN, ViT, etc. For deep learning models that are good at sequence information processing, the number of bands in multispectral images may be too small compared with hyperspectral images, which is not conducive to the performance of 1D CNN, resulting in the non-optimal performance of 1D CNN. CNN is different, while RNN is designed for sequence data, and the multispectral image classification performance is significantly altered by 1D CNN compared with 1D CNN, but RNN struggled to learn long-term dependencies. While Transformer can capture global sequence information via location encoding, except for data from the Nanxun district, for the rest of the dataset, Transformer’s multispectral image classification performance is stronger than that of RNN. The sequence dimension of multispectral images is enhanced by the fully connected layer, and then reshaped into a 3D feature map for 2D CNN classification, transforming sequence classification into a similar mode of image classification. The performance of FC + 2D CNN is slightly better than that of Transformer, and the structure of 2D CNN developed in this paper is simpler, while its training time is shorter than that of Transformer. Increasing the depth of the network may help to improve the performance of FC + 2D CNN, and it may be better to convert 1D sequences into 2D feature maps using mathematical methods such as Grammy angle fields and Markov variation fields. Although HyFormer is a network designed for multispectral images and has not been experimented with panchromatic and hyperspectral images, it may also provide new ideas for the classification of panchromatic and hyperspectral images, and may have better results for these two types of images. Using HyFormer with FC + 2D CNN to extract features at different levels of pixels with raw spectral information as the Transformer input performs best when applied to all three years of the dataset. This is because the combination of features extracted by CNN with the original waveform information to achieve multi-feature fusion is also benefited by Transformer’s processing of sequence information, which further optimizes the expressiveness of the input features, resulting in better classification results.

## 5. Conclusions

In this paper, we focused on fusing the features of 2D CNN and Transformer to achieve the pixel sequence classification of multispectral images. Two-dimensional convolutional neural networks often handle pixel classification by extracting the neighboring blocks of target pixels as the input, which cannot achieve pixel-level accuracy; in this paper, we designed a network structure of FC + 2D CNN to solve the problem that 2D CNN cannot classify pixel sequences. In classification, which is performed in order to further enhance the expressions of sequence information features, the features of different levels of 2D CNN are extracted and fused with the original waveform information as the input of Transformer, and shallow information is passed to a deeper level by inserting skip connections between adjacent Transformer Encoders to reduce the information loss. In the future, we will further improve the conversion of 1D pixel sequences to 2D or 3D feature maps to enhance the diversity of features, and make this model more suitable for multispectral image classification. In addition, reducing the complexity of Transformer while maintaining its high performance is also an area of concern. Traditional methods sometimes add vegetation indices, building indices and other remote sensing indices (obtained through RGB and NIR band calculations) to the original bands for the purpose of improving the differentiation between groups, which may also be effective. In conclusion, further improving the classification effect of 2D CNN and Transformer on pixel sequences will be the focus of our future work.

## Figures and Tables

**Table 1:** Comparison of the classification results of different classification methods on the study area 2021 dataset.

NO.	Land-Cover Type	Changxing			Nanxun		
		Training	Validation	Testing	Training	Validation	Testing
1	Build-up	1956	652	652	2515	838	839
2	Water	2016	672	673	1878	626	627
3	Tree	2119	706	707	2531	844	844
4	Pond	610	204	204	1289	430	430
5	Rice	835	278	279	668	223	223
6	Othercrop	478	159	160	549	183	183
7	Plantation	819	273	273	1114	371	372
8	Greenhouse	558	186	186	411	137	138
	Total	9391	3130	3134	10,955	3652	3656

**Table 2:** Multi-method parameters.

Algorithms	Parameters
SVM	kernel = “rbf”, decision_function_shape = “ovr”, C=10
1D CNN	Conv1d(1, 20, kernel_size = 1, stride = 1), MaxPool1d(4, stride = 1, padding = 0)
RNN	two gated recurrent units
Transformer	depth = 5, dim = 64, heads = 4, dropout = 0.1
SpectralFormer	depth = 5, dim = 64, heads = 4, dropout = 0.1, GSE = 2
FC + 2D CNN	conv2d(in_channel = 16, out_channel = 64, kernel_size = 1, stride = 1)conv2d(in_channel = 64, out_channel = 64, kernel_size = 3, stride = 1)AvgPool2d(kernel_size = 2, stride = 2, padding = 0)
HyFormer	depth = 5, dim = 64, heads = 4, dropout = 0.1

**Table 3:** Test set results for the study area at different learning rates.

Class No.	Learning Rate		
	0.05	0.005	0.0005
1	96.78	96.01	97.39
2	99.11	99.11	99.85
3	95.61	94.90	94.33
4	95.09	96.57	97.06
5	89.92	92.09	92.80
6	74.84	80.50	81.76
7	86.08	91.94	92.67
8	90.86	87.63	93.55
OA(%)	93.90	94.47	95.37
AA(%)	91.04	92.34	93.68
Kappa	92.6700	0.9337	0.9444

**Table 4:** Results of ablation experiments on the study area’s testing set using HyFormer with different module combinations.

Method	Module		Metric		
	FC + CNN	SC	OA(%)	AA(%)	Kappa
Transformers(ViT)	**×**	**×**	94.15	92.61	0.9300
FC + CNN	**×**	**×**	94.41	92.47	0.9330
HyFormer	✓	**×**	95.27	93.43	0.9432
HyFormer	**×**	✓	94.25	92.23	0.9311
HyFormer	✓	✓	**95.37**	**93.68**	**0.9444**

**Table 5:** HyFormer test results using different training sample proportions in the 2022 Changxing County testing set.

Ratio	Class No.								Metric		
	1	2	3	4	5	6	7	8	OA(%)	AA(%)	Kappa
10%	97.85	99.01	97.73	93.07	73.38	86.07	79.41	89.09	92.62	89.20	0.9112
20%	94.47	99.17	96.46	94.09	82.01	91.82	80.57	91.40	93.15	91.25	0.9179
40%	97.47	99.59	97.02	95.58	92.08	89.93	93.95	95.43	96.35	95.13	0.9562

**Table 6:** Comparison of classification results of different classification methods applied on the testing set of Changxing County, 2022.

Class No.	Method						
	SVM	1D CNN	RNN	Transformer (ViT)	SpectralFormer	FC + 2D CNN	HyFormer
1	92.58	92.48	94.78	94.32	95.24	95.52	**97.39**
2	98.21	90.32	99.70	99.55	98.96	99.55	**99.85**
3	**98.11**	88.67	94.05	92.77	94.90	94.47	94.33
4	88.19	74.02	96.08	**97.06**	**97.06**	95.58	**97.06**
5	84.43	38.13	90.65	**93.52**	**93.52**	89.21	92.80
6	56.06	2.51	72.32	77.99	74.21	77.36	**81.76**
7	76.55	59.71	87.54	**92.67**	85.71	92.30	**92.67**
8	67.92	5.37	94.08	93.01	92.47	**95.69**	93.55
OA(%)	89.31	72.52	93.58	94.15	93.87	94.41	**95.37**
AA(%)	82.76	56.4	91.15	92.61	91.51	92.47	**93.68**
Kappa	0.8711	0.6652	0.9230	0.9300	0.9264	0.9330	**0.9444**
Times(s)	/	463	837	3067	5223	903	4733
Params	/	209	585	89,841	90,006	81,033	171,153

**Table 7:** Comparison of classification results of different classification methods applied on the testing set of Nanxun District, 2022.

Class No.	Method						
	SVM	1D CNN	RNN	Transformer (ViT)	SpectralFormer	FC + 2D CNN	HyFormer
1	95.35	99.64	99.88	99.76	99.88	**100.00**	99.40
2	**100.00**	96.80	**100.00**	99.52	**100.00**	**100.00**	99.84
3	**100.00**	**97.03**	92.53	92.18	93.12	95.61	94.79
4	94.88	96.51	99.76	**100.00**	**100.00**	**100.00**	**100.00**
5	0.00	0.00	57.84	55.60	58.29	55.15	**63.21**
6	0.00	42.07	94.53	94.53	94.53	91.25	**95.63**
7	55.37	83.55	96.22	96.22	**96.49**	93.53	92.45
8	97.10	0.00	**100.00**	**100.00**	**100.00**	**100.00**	**100.00**
OA(%)	82.58	83.34	94.99	94.69	95.21	95.15	**95.40**
AA(%)	67.84	64.45	91.15	92.23	92.79	91.95	**93.17**
Kappa	0.7865	0.8008	0.9230	0.9361	0.9423	0.9415	**0.9446**

**Table 8:** Comparison of the classification results of different classification methods on the dataset after adding red edge bands.

Class No.	Method						
	SVM	1D CNN	RNN	Transformer (ViT)	SpectralFormer	FC + 2D CNN	HyFormer
1	95.65	91.56	97.08	97.08	**98.16**	97.70	**98.16**
2	**100.00**	97.47	99.70	99.70	99.85	99.70	99.25
3	**99.43**	96.45	96.74	97.45	98.58	98.16	98.58
4	64.02	88.72	**97.06**	96.57	96.57	96.08	96.57
5	87.64	92.8	99.64	99.64	99.64	99.64	**100.00**
6	22.50	33.96	82.38	86.16	**90.56**	86.16	88.68
7	60.44	62.27	90.11	93.77	94.14	93.40	**97.07**
8	40.32	55.37	94.62	97.31	97.31	95.70	**97.85**
OA(%)	85.43	86.23	96.29	97.09	97.86	97.22	**97.96**
AA(%)	71.25	77.33	94.67	95.96	96.85	95.82	**97.02**
Kappa	0.821	0.8342	0.9555	0.9651	0.9743	0.9667	**0.9755**

**Table 9:** Comparison of the classification results of different classification methods on the dataset after adding SWIR bands.

Class No.	Method						
	SVM	1D CNN	RNN	Transformer (ViT)	SpectralFormer	FC + 2D CNN	HyFormer
1	95.98	97.08	96.16	97.54	98.62	**99.23**	**99.23**
2	**100.00**	98.06	99.55	99.70	99.70	98.81	99.70
3	97.03	99.29	97.73	99.71	99.29	**99.86**	99.57
4	76.89	85.78	95.58	95.59	98.53	**99.51**	98.04
5	99.44	15.82	98.56	98.20	98.92	**99.64**	98.56
6	16.87	0.63	88.68	95.59	**96.22**	90.56	95.60
7	93.04	51.65	89.74	97.06	**99.63**	97.43	98.17
8	45.70	6.98	91.39	96.77	97.31	97.31	**98.39**
OA(%)	89.74	75.62	96.20	98.24	98.91	98.63	**98.95**
AA(%)	78.12	56.92	94.68	97.52	98.53	97.80	**98.41**
Kappa	0.875	0.6979	0.9544	0.9789	0.9870	0.9835	**0.9874**

**Table 10:** Sample size of dataset in Changxing County in 2018 and 2020.

NO.	Land-Cover Type	2018			2020		
		Training	Validation	Testing	Training	Validation	Testing
1	Bulidup	1814	605	605	1863	621	622
2	Water	2463	821	821	3186	1062	1063
3	Tree	2119	706	707	2119	706	707
4	Pond	738	246	247	907	303	303
5	Rice	1132	377	378	1066	355	356
6	Othercrop	364	121	122	478	159	160
7	Plantation	819	273	273	819	273	273
8	Greenhouse	558	186	186	558	186	186
	Total	10,007	3335	3339	10,996	3665	3670

**Table 11:** Comparison of classification results of different classification methods applied on the testing set of Changxing County, 2018.

Class No.	Method						
	SVM	1D CNN	RNN	Transformer (ViT)	SpectralFormer	FC + 2D CNN	HyFormer
1	92.89	75.04	94.21	95.87	96.03	96.03	**97.19**
2	**100.00**	89.16	99.75	99.88	99.75	99.87	99.63
3	**99.29**	99.01	97.59	96.88	97.87	98.30	98.44
4	98.38	79.27	98.78	98.37	98.37	**98.78**	**98.78**
5	94.18	61.8	**94.43**	91.51	91.51	85.94	91.78
6	6.56	8.26	38.84	60.33	59.50	**76.85**	75.21
7	45.42	5.86	58.61	63.37	73.62	72.89	**75.09**
8	91.93	32.26	94.09	94.09	**95.70**	95.69	**95.70**
OA(%)	89.46	71.93	91.72	92.71	93.82	93.91	**94.87**
AA(%)	78.58	56.33	84.54	87.54	89.05	90.55	**91.48**
Kappa	0.8727	0.6558	0.9005	0.9124	0.9257	0.9269	**0.9384**

**Table 12:** Comparison of classification results of different classification methods applied on the testing set of Changxing County, 2020.

Class No.	Method						
	SVM	1D CNN	RNN	Transformer (ViT)	SpectralFormer	FC + 2D CNN	HyFormer
1	97.10	84.05	98.55	98.06	98.55	**98.88**	98.06
2	**100.00**	86.06	99.81	**100.00**	**100.00**	**100.00**	**100.00**
3	94.15	**99.71**	94.19	95.61	94.90	96.88	98.16
4	92.73	59.73	96.69	**98.35**	98.01	97.69	96.70
5	67.73	22.25	87.88	**91.83**	90.42	89.29	87.89
6	70.00	66..03	74.84	83.64	**93.08**	84.27	91.94
7	70.69	15.75	79.48	77.65	76.55	77.65	**83.88**
8	91.93	25.8	91.93	95.16	96.77	**97.31**	96.77
OA(%)	90.68	70.83	94.11	95.28	95.47	95.50	**96.15**
AA(%)	85.46	57.43	90.43	92.54	93.54	92.75	**94.10**
Kappa	0.8866	0.6398	0.9284	0.9427	0.945	0.9453	**0.9532**

**Table 13:** Table of land use type changes in the study area for different periods from 2018 to 2022.

Class	Area (km^2^)			Area Change Rate (%)		
	2018	2020	2022	2018–2020	2020–2022	2018–2022
Buildup	66.2185	65.9379	63.0991	−0.42	−4.31	−4.71
Water	52.5349	51.8713	52.5555	−1.26	1.32	0.04
Tree	34.3652	39.6366	35.7494	15.34	−9.81	4.03
Pond	10.6323	13.3764	8.3785	25.81	−37.36	−21.20
Rice	32.6588	21.5091	25.3542	−34.14	17.88	−22.37
Othercrop	15.7600	13.2754	9.6773	−15.77	−27.10	−38.60
Plantation	16.6443	23.0769	28.6343	38.65	24.08	72.04
Greenhouse	8.9834	9.1138	14.3491	1.45	57.44	59.73

## Data Availability

The code will be available at https://github.com/Yancccccc/HyFormer accessed on 1 November 2022.

## Abbreviations

The following abbreviations are used in this manuscript:

RS	Remote Sensing
CNN	Convolutional Neural Network
FC	Fully Connected Layer
ViT	Vision Transformer
SVM	Support Vector Machine
MLE	Maximum Likelihood Method
RF	Random Forest
KNN	K-Nearest Neighbor
NLP	Natural Language Processing
SE	Squeeze-and-Excitation
SC	Skip connection
